# GM-CSF and the role of myeloid regulatory cells in the pathogenesis and treatment of Crohn’s disease

**DOI:** 10.1186/s40348-015-0024-4

**Published:** 2015-12-01

**Authors:** Jan Däbritz

**Affiliations:** Present address: Department of Pediatrics, University Hospital Rostock, Ernst-Heydemann-Str. 8, 18057 Rostock, Germany; Department of Pediatric Rheumatology and Immunology, University Hospital Münster, Albert-Schweitzer-Campus 1, 48149 Münster, Germany; Murdoch Children’s Research Institute, The Royal Children’s Hospital Melbourne, 50 Flemington Road, Parkville, VIC 3052 Australia

## Abstract

**Background:**

Intestinal monocytes/macrophages sustain the intestinal immune homeostasis and might be an attractive therapeutic target for the management of inflammatory bowel disease (IBD). Granulocyte macrophage colony-stimulating factor (GM-CSF) exerts beneficial effects on intestinal inflammation and promotes signal transducer and activator of transcription 3 (STAT3)-mediated expansion of myeloid-derived suppressor cells (MDSCs). However, the full action mechanism of GM-CSF, and especially whether monocytes mediate its therapeutic effects in vivo, had not been previously elucidated.

**Conclusions:**

This review article summarizes recent developments in the immunology of mucosal diseases and describes new aspects of the role of myeloid regulatory cells in IBD and the function of GM-CSF in maintaining the intestinal immune homeostasis in Crohn’s disease (CD). This review article highlights the exploration of stimulating in addition to suppressive therapies for patients with IBD and underpins that myeloid regulatory cells might become a promising novel cell-based therapeutic option.

## Introduction

**Fig. 1:**
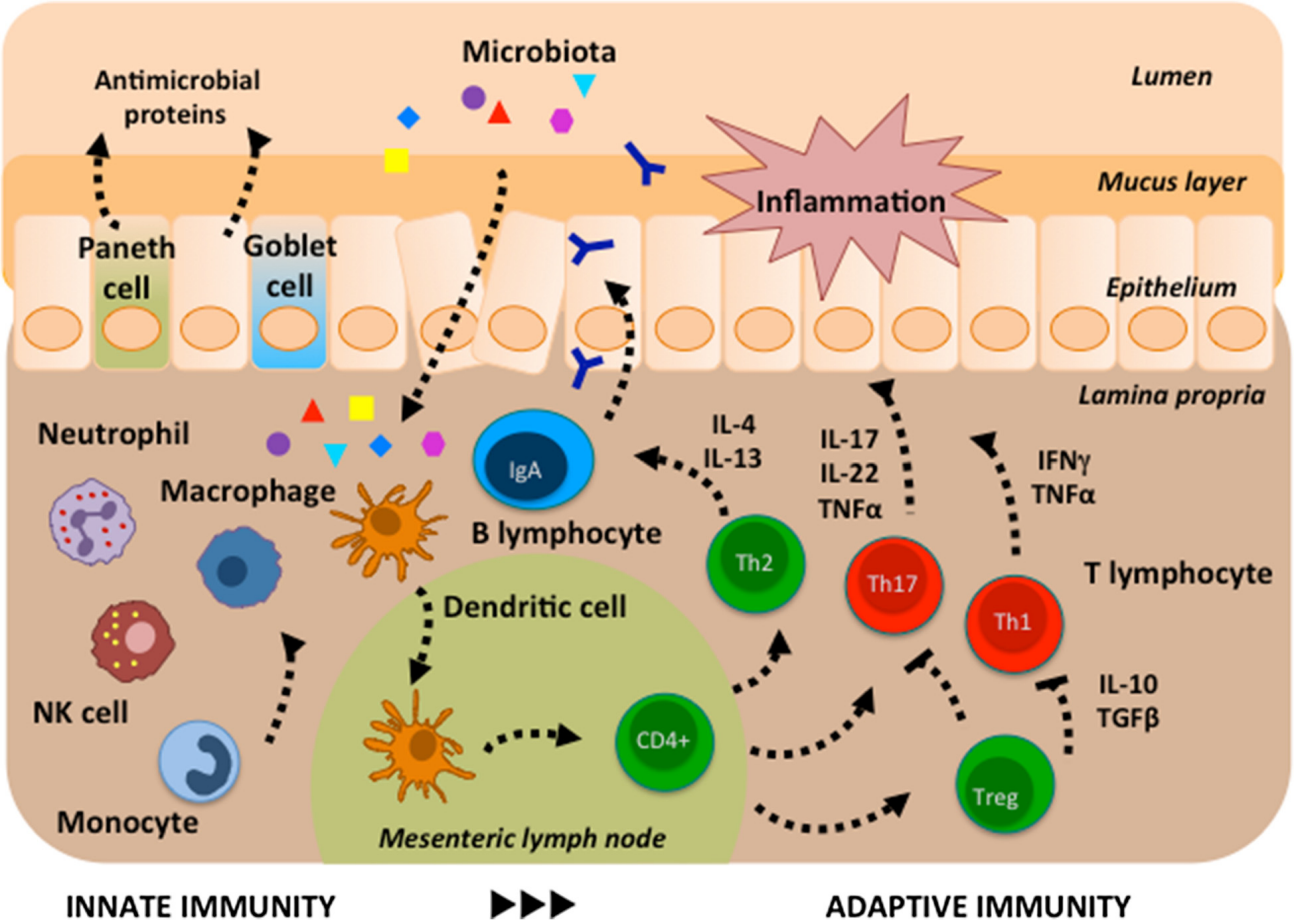
Overview of the intestinal immune system. Innate immunity: Intestinal epithelial cells provide a physical barrier between the luminal microbes and the underlying intestinal tissues to control defense and tolerance. Specialized epithelial cells produce a mucus layer (goblet cells) and secrete antimicrobial proteins (Paneth cells) that limit bacterial exposure to the epithelial cells. Production of large amounts of immunoglobulin (Ig) A provides additional protection from luminal microbiota. Innate microbial sensing by epithelial cells, dendritic cells, and macrophages is mediated through pattern recognition receptors and induces various pathways that mediate microbial killing and activate adaptive immune cells. Adaptive immunity: Dendritic cells present antigens to naïve CD4 positive (+) T cells in secondary lymphoid organs (mesenteric lymph nodes), where factors such as the phenotype of the antigen-presenting cells and cytokine milieu modulate differentiation of proinflammatory T helper (Th) cell subsets (Th1, Th17) with characteristic intestinal homing profiles and cytokines such as interleukin (IL)-17, IL-22, tumor necrosis factor alpha (TNFα), and interferon gamma (IFNγ). Defense mechanisms that limit microbial entry into intestinal tissues also serve as a mechanism of tolerance. Activation of unique populations of dendritic cells in the intestinal lamina propria does not result in secretion of proinflammatory cytokines. Those dendritic cells present antigen to T cells in mesenteric lymph nodes, which leads to differentiation of regulatory (Treg) and anti-inflammatory (Th2) T cell populations, mediated by transforming growth factor beta (TGFß), IL-4, IL-10, and IL-13

Crohn’s disease (CD) is a chronic relapsing disease and is a manifestation of a dysregulated immune response against the microorganisms of the intestinal flora in genetically susceptible individuals [1]. Various components of the mucosal immune system are implicated in the pathogenesis of inflammatory bowel diseases (IBD) and include intestinal epithelial cells; innate lymphoid cells; cells of the innate immune system such as monocytes, macrophages, neutrophils, and dendritic cells (DCs); the adaptive immune system with T and B cells; and their secretion products (chemokines, cytokines) (Fig. 1). Investigating the microbiome will come up with new theories on the etiology of IBD. Thus, the increasing knowledge of the interaction between the innate and adaptive immune systems as well as the microbiome in the context of the individual genetic background might be helpful for personalized therapy in IBD in the future. Indeed, the intestinal mucosa is host to a massive number of intestinal macrophages resident in the mucosa which continuously demonstrate phagocytic activity, and blood monocytes are recruited to the lamina propria, normally quickly undergo induction of anergy but interestingly retain many proinflammatory activities in inflamed mucosa. Although the exact cause of CD remains unknown, new pathogenic paradigms in IBD have highlighted that the interactions between various constituents of the innate and adaptive immune systems play key roles in the pathogenesis of IBD [2]. As the frontiers of immunological research expand, new insights into the pathogenesis of IBD are opening up new possible avenues for treatment. Myeloid-derived cells including monocytes/macrophages long thought to be effector cells driving the initiation of inflammation have been increasingly shown to have immunoregulatory effects previously under-appreciated. Recent work suggests an important protective role of monocytes/macrophages and possible homeostatic mechanisms to restrain acute and chronic intestinal inflammation (reviewed in [3]). This brief review highlights the important role of immunoregulatory monocytes in IBD and the role of granulocyte macrophage colony-stimulating factor (GM-CSF) in maintaining the intestinal immune homeostasis in CD. For earlier and more detailed literature on the immunoregulatory role of myeloid-derived cells in IBD, the reader is referred to prior reviews ([3] and references cited therein).

## Role of monocytes/macrophages and GM-CSF in the immunology of mucosal diseases

Monocytes and their derivative cells play an important role in the pathophysiology of IBD. Intestinal macrophages derived from blood monocytes play a key role in sustaining the innate immune homeostasis in the intestine [4]. Intestinal chronic inflammation may be caused by inborn errors of macrophages in patients with CD. Macrophages from CD patients are intrinsically defective, with impaired secretion of cytokines that are normally translated but internally degraded. Because of insufficient production of cytokines and chemokines, there is impaired attraction of granulocytes to mucosal breaches. Impaired acute, granulocytic inflammation results in impaired clearance of bacteria and debris from the gut wall, itself resulting in chronic, granulomatous inflammation [5, 6]. The cytokine GM-CSF promotes the differentiation of monocytes into macrophages and DCs. There is growing evidence that GM-CSF, or rather the lack thereof, is implicated in the pathogenesis of CD. Human and murine studies showed that GM-CSF exerts beneficial effects on intestinal inflammation. In addition, it has been shown that increased levels of neutralizing endogenous serum GM-CSF autoantibodies (Ab) in CD patients as well as decreased expression of the GM-CSF receptor α-subunit (CD116) in circulating monocytes and granulocytes of CD patients affect mucosal integrity, epithelial barrier function, bacterial killing, and neutrophil antimicrobial functions (reviewed in [7]).

GM-CSF also triggers several different signaling pathways in myeloid-derived suppressor cells (MDSCs) that mainly involve the signal transducer and activator of transcription (STAT) family of transcription factors. MDSCs are a heterogeneous group of immature cells that includes precursors of macrophages, granulocytes, DCs, and myeloid cells at earlier stages of differentiation. MDSCs expand rapidly during inflammation, infection, and cancer and suppress T cell responses [8]. Although MDSCs have been described in animal models of experimental colitis and in patients with IBD, their exact role in IBD pathogenesis is unclear [9]. As an additional homeostatic function, GM-CSF has been proposed to be essential for a microbiota-dependent crosstalk between mononuclear phagocytes and group 3 innate lymphoid cells (ILC3s) thereby promoting intestinal homeostasis. ILC3-derived GM-CSF can promote intestinal myeloid cell homeostasis through enhancing DC and regulatory T cell function [10–13].

The potential for future cell-based therapies for IBD is enhanced by the advances being made in the understanding of the innate immune system in the intestine. Based on this, any therapeutic approach aimed at modulating monocytes/macrophages as a key player in innate immunity could be a potent way to modulate/manage intestinal inflammation in patients with chronic intestinal inflammation. However, the full action mechanism of GM-CSF, and especially whether monocytes mediate its therapeutic effects *in vivo*, had not been previously elucidated.

## Human GM-CSF-activated monocytes have regulatory immune functions in vitro

We hypothesized that GM-CSF might activate monocytes in a way that modulates their function during intestinal inflammation. We analyzed the immunoregulatory potential of GM-CSF on human monocytes *in vitro* using microarray technology and functional assays taking all potential functions of human GM-CSF-activated monocytes into account (including gene expression, innate immune functions, interplay with adaptive immunity, wound healing). Our findings suggest that the early imprinting of monocytes after activation with GM-CSF is of crucial importance, because monocytes play an important role during the recruitment phase of the innate immune response and have the potential to regulate adaptive immune mechanisms.

We showed that GM-CSF-activated monocytes combine (i) an anti-inflammatory phenotype, (ii) features of augmented innate immune functions (e.g., migration, chemotaxis, oxidative burst), (iii) the ability to facilitate epithelial healing, and (iv) the regulatory potential on adaptive immunity. GM-CSF-activated monocytes represent an intermediate cell type, combining cell-surface expression characteristics and functional features of different M2-type macrophage subsets [14]. In contrast with proinflammatory and antimicrobial responses of classically activated M1-like monocytes, M2-like phenotypes are broadly anti-inflammatory and play important roles in wound healing [15]. In addition to these innate immune functions, human GM-CSF-activated monocytes simultaneously have a regulatory potential on adaptive immunity. We showed that GM-CSF significantly induces a short-termed expression of chemokines in monocytes, which are known to attract naïve T cells, T helper 2 (Th2) cells, and/or regulatory T cells (Treg). Indeed, migration of naïve, autologous T cells toward GM-CSF-activated monocytes was accelerated in vitro. Our data suggest that particularly CCL18 and CCL23 might be responsible for the increased T cell migration. When we cocultured GM-CSF-activated monocytes with syngeneic CD4^+^ T cells, we observed that GM-CSF-activated monocytes attract T cells and shape their differentiation toward Th2 cells by upregulating T cell-derived IL-4, IL-10, and IL-13 and downregulating T cell-derived IFNγ *in vitro*. In addition, cocultures of GM-CSF-activated monocytes and naïve T cells led to an induction of Treg, which produce anti-inflammatory IL-10 and TGFβ and have important anti-inflammatory functions [14].

Finally, we showed that GM-CSF may regulate the homing molecules CCR2 and CCR6 on human monocytes [14], which are involved in regulating several aspects of mucosal immunity, including the ability to mediate the recruitment of innate immune cells to the sites of epithelial inflammation.

## Murine GM-CSF-activated monocytes as an anti-inflammatory player in the intestine

After having analyzed the specific activation pattern of human monocytes in response to GM-CSF *in vitro*, we next aimed to address the functionality of these cells *in vivo* in the context of CD. Because systematic analyses in the human system are not feasible, we went on analyzing the effects of GM-CSF-activated monocytes on the murine system. In the first step, we activated bone-marrow-derived murine monocytes with GM-CSF *in vitro* and analyzed the phenotype and functional properties of murine GM-CSF-activated monocytes *in vitro*.

Our gene expression analyses showed that murine GM-CSF-activated monocytes have a similar gene regulation profile when compared with the GM-CSF-dependent gene expression in human monocytes [14]. Like human GM-CSF-activated monocytes, murine GM-CSF-activated monocytes did not fit into current monocyte subtype definitions dividing monocytes/macrophages according to cell surface markers and functions in classic/inflammatory (M1), intermediate, and alternative/anti-inflammatory (M2) cells. After activation with GM-CSF, murine monocytes were found to overexpress some surface markers of specialized macrophages and some stimulatory molecules while showing decreased expression of a marker for alternatively activated monocytes. In addition, we noted discrepancies in the expression levels of some M2-polarized markers. Because this expression profile did not fully recapitulate the previously reported activation stages of monocytes, we proposed that the newly generated monocytes represented a unique and distinct state of activation. These murine GM-CSF-activated monocytes were also found to be somewhat unique from a functional point of view. We demonstrated *in vitro* that this population has a lower capacity for phagocytosis and adhesion but triggers increased generation of reactive oxygen species, which are required for pathogen clearance. Moreover, after lipopolysaccharide treatment, proinflammatory cytokine production was found to be higher in GM-CSF-activated monocytes compared with untreated monocytes. However, despite their phenotypical and functional diversity, murine GM-CSF-activated monocytes mainly seem to represent monocytes in a developmental stage passing toward M2-polarized macrophages with an upregulation of M2 markers and important anti-inflammatory functions [16].

In addition, we performed flow cytometry-based staining of various gut homing molecules on monocytes and found, in agreement with our studies on human GM-CSF-activated monocytes, CCR2, necessary for migration toward inflammatory sites, and CCR6, used for the migration toward lymph follicles such as Peyer patches, to be upregulated on murine GM-CSF-activated monocytes [16].

## Murine GM-CSF-activated monocytes reduce severity of experimental colitis

In the next step, we tested whether GM-CSF-activated monocytes have a protective and therapeutic effect against intestinal inflammation in vivo. Since none of the currently available animal models of IBD reflects all aspects of IBD in humans, we used various mouse models of chronic colitis. Firstly, we showed that Rag1-deficient mice (which lack all mature adaptive immune cells) were protected from disease progression during CD4^+^CD25^−^ T cell-induced experimental colitis when they received GM-CSF-activated monocytes but not untreated monocytes. In agreement with earlier work, we found that Rag1-deficient mice that did not receive GM-CSF-activated monocytes but GM-CSF intraperitoneally after the transfer of T cells were not completely protected from colitis but showed reduced disease severity. Our data demonstrate that the protective effect of monocytes depends on their GM-CSF pre-activation in a T cell-dependent model of colitis [14]. Secondly, we induced chronic colitis in wild-type mice by repetitive administration of dextran sulfate sodium (DSS) in drinking water. Mice treated with GM-CSF-activated monocytes were again completely protected from experimental colitis [16]. Thirdly, we induced chronic colitis in Rag1-deficient mice with DSS. Injection of GM-CSF-activated monocytes was not able to protect Rag1-deficient mice against DSS-induced colitis, indicating that adaptive immunity is required for the protection mediated by GM-CSF-activated monocytes [16].

We next sought to decipher the mechanism by which GM-CSF-activated monocytes confer their therapeutic effects on intestinal inflammation. We found that, compared with control monocytes, the GM-CSF-activated monocytes were taken up faster by the inflamed intestine and stayed there longer, especially in Peyer patches [14, 16]. In addition to these unique migratory features and in agreement with our in vitro findings, accelerated gut homing of GM-CSF-activated monocytes was accompanied by increased production of the anti-inflammatory Th2 cytokines IL-4, IL-10, and IL-13 and decreased production of the proinflammatory Th1 cytokine IFNγ in intestinal lamina propria mononuclear cells and mesenteric lymph nodes in vivo during colitis [14]. Furthermore, GM-CSF-activated monocytes increased the accumulation of Foxp3^+^ Tregs in the lymph follicles of mice with experimental colitis when compared with untreated mice or those treated with nonactivated monocytes. In an in vitro model in which GM-CSF-activated monocytes were cocultured with naïve T cells, we observed increased proliferation and differentiation of Foxp3^+^ Treg cells and found that this occurred through a mechanism that involves the CD39-mediated conversion of ATP into adenosine—a mechanism that was previously reported to induce Treg cell differentiation [16].

**Fig. 2:**
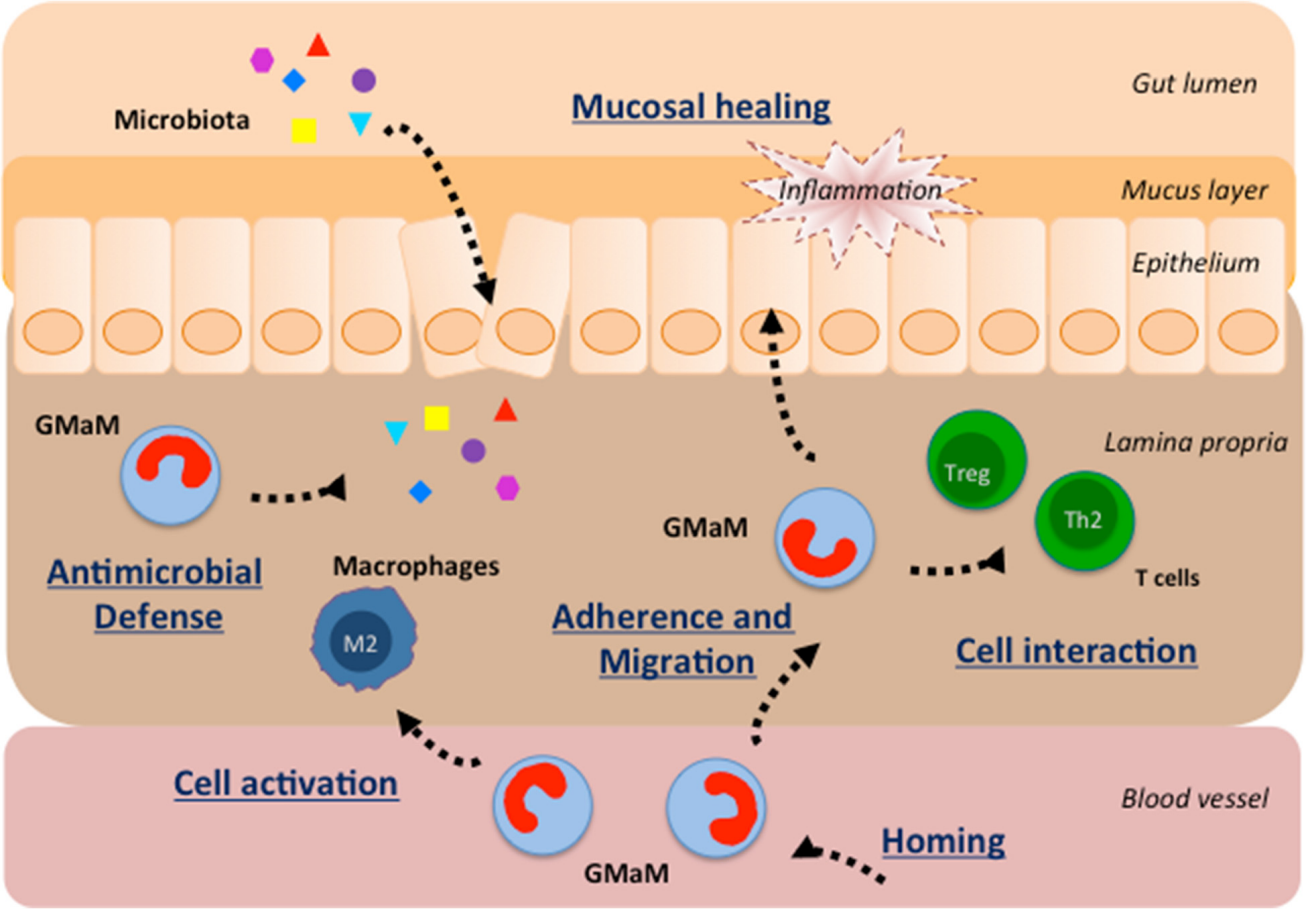
Mechanisms of immunoregulation by GMaM in inflammatory bowel disease. The early imprinting of monocytes following activation with GM-CSF is of crucial importance, since monocytes play an important role during the early (recruitment) phase of the innate immune response in the intestine. GMaM represent a unique and distinct state of activation combining phenotypical and functional features of different M2 macrophage subsets. GMaM trigger augmented host defense functions including an increased generation of reactive oxygen species, which are required for pathogen clearance. GMaM have a higher capacity for adherence, migration, and chemotaxis; they attract T cells and have the potential to regulate adaptive immune mechanisms by induction of anti-inflammatory T helper 2 cells (Th2) and regulatory T cells (Treg). Furthermore, GMaM have the ability to facilitate epithelial/mucosal healing and to ameliorate intestinal inflammation. *GMaM* granulocyte macrophage colony-stimulating factor (GM-CSF)-activated monocytes

Taken together, these important data show that GM-CSF-activated monocytes are a specific population of monocytes that has a protective effect against intestinal inflammation (Fig. 2). The beneficial effects of GM-CSF-activated monocytes are T cell dependent and involve the CD39/adenosine-mediated proliferation and differentiation of Foxp3^+^ Treg cells. This is in agreement with the work of others, who showed that GM-CSF can influence the interactions between DCs and T cells and in this manner determine a pro- versus anti-inflammatory adaptive immune response via Th1 and Th17 cell responses or the induction of Treg cells, respectively [17–20].

## Neutralization of GM-CSF is associated with disease relapse in CD patients

GM-CSF bioactivity varies inversely with increasing serum levels of endogenous neutralizing GM-CSF autoantibodies (Ab). GM-CSF Ab as well as defective GM-CSF receptor expression have been described in IBD [21–25]. Increased GM-CSF Ab levels are associated with alterations of epithelial barrier function, bowel permeability, bacterial translocation, neutrophil antimicrobial functions, and epithelial cell survival and proliferation [7, 21–24, 26, 27]. A rare familial form of pulmonary alveolar proteinoses may also serve as an established model to highlight the role of Ab toward GM-CSF resulting in blocking GM-CSF-driven responses [28, 29]. This suggested that variation in GM-CSF Ab and thereby GM-CSF bioactivity might also be associated with clinical disease relapses in patients with CD. In order to validate these findings, we determined GM-CSF Ab levels in prospectively collected serum samples of pediatric and adult patients with CD. Time course analysis of GM-CSF Ab up to 9 months before and after relapse showed a clear increase of Ab titers up to 6 months before clinical relapse followed by a steady decrease, likely indicating the success of the intensified therapies. In addition, we found that GM-CSF Ab levels in serum samples of patients with IBD who had no clinical disease relapse during the study follow-up showed a low intra-individual variation and GM-CSF Ab levels were below the cut-point where GM-CSF Ab start to inhibit neutrophil antibacterial function [30]. Thus, an elevated serum GM-CSF Ab titer in patients with CD in clinical remission as defined by clinical disease activity scores may represent an early stage of increased intestinal permeability, bacterial translocation, neutrophil dysfunction, and reduced antimicrobial activity (reviewed in [31]). It is conceivable that the potentially impaired intestinal homeostasis progresses to cause an eventual clinical relapse of the disease and underpins the beneficial role of GM-CSF in the context of intestinal homeostasis.

## Blood monocytes from patients with CD behave like GM-CSF-activated monocytes

Our phenotypic and functional studies showed that peripheral blood monocytes from CD patients in clinical remission are not impaired compared to healthy controls [32]. We next studied phenotypic and functional features of untreated versus GM-CSF-activated peripheral blood monocytes of patients with quiescent CD. Collectively, our data suggest that the effects of GM-CSF activation of peripheral blood monocytes from patients with CD are similar to the observed effects of GM-CSF-activated monocytes from healthy donors. This included the GM-CSF-induced increase in adherence, migration, chemotaxis, and oxidative burst, as well as the priming of monocytes to secondary microbial stimuli. In addition, changes in GM-CSF-dependent mRNA expression of selected key inflammatory cytokines were in agreement with our transcriptomic data obtained from GM-CSF-activated monocytes of healthy individuals. Importantly, there was no evidence that GM-CSF activation had different effects on monocytes when compared between individual CD patients [14].

## Myeloid-specific STAT3 activation ameliorates experimental colitis via MDSCs

Several studies have investigated the mechanisms of MDSC T cell suppressive activity in health and disease including experimental colitis models and human IBD [8, 9]. MDSC expansion is regulated by several factors (including GM-CSF) that are released during inflammation, infection, and cancer. The signaling pathways in MDSCs converge on Janus kinase (JAK) protein family members and STAT3. Hyperactivation of STAT3 in gp130^757F/F^ mice is associated with protection from DSS-induced colitis. We hypothesized that the protective role of gp130-dependent STAT3 activation in experimental IBD involves the expansion and activation of MDSCs, in addition to the previously reported proliferative, regenerative, and survival effects on intestinal epithelial cells. We therefore induced acute and also chronic colitis with DSS and analyzed the effects in mice with systemic hyperactivation of STAT3 (gp130^757F/F^) ± myeloid-specific STAT3-deficiency (LysMcre/STAT3^flox^). We showed that the resistance to DSS-induced acute colitis in gp130^757F/F^ mice is via myeloid cell-specific STAT3 activation, expansion of granulocytic MDSCs in the colon, and increased production of suppressive and protective mediators. Interestingly, gp130^757F/F^ mice were not resistant to chronic DSS-induced colitis [33].

Thus, our study identified new immunoregulatory mechanisms of STAT3 during intestinal inflammation. Importantly, the adoptive transfer of MDSCs in different animal models of IBD ameliorates colitis and suggests that MDSCs may be used as the basis for a novel adoptive cellular cell therapy in IBD ([3] and references cited therein). However, MDSCs may either promote or suppress T cell functions depending on the stage of disease and/or other external factors. Indeed, there is also evidence supporting a proinflammatory role of myeloid cells in experimental IBD [34–36]. In addition, an increase in the frequency of MDSCs in the peripheral blood from patients with active chronic colitis has been reported [37].

## Summary and future perspectives

The work of others and our in vitro and in vivo data show that GM-CSF-activated monocytes are a specific population of monocytes that has a protective effect against intestinal inflammation as shown in various independent mouse models of experimental colitis. The beneficial effects of GM-CSF-activated monocytes are T cell dependent and involve the proliferation and differentiation of Th2 and Treg cells in the intestine (Fig. 2). Thus, our in vitro and in vivo findings support the exploration of stimulating rather than suppressive therapies with the potential to more specifically reprogram monocytes toward immunoregulatory functions to alleviate chronic CD. Even though previous clinical trials showed that systemic administration of GM-CSF might have therapeutic effects in a subgroup of patients with CD (reviewed in [38]), it is important to consider that key aspects of GM-CSF biology and unwanted side effects of systemic administration still need to be clarified [39]. Adverse events commonly associated with systemic GM-CSF administration include injection site reactions, bone and musculoskeletal chest pain, pulmonary capillary leak syndrome, pulmonary edema, heart failure, fever, and neurotoxicity. In addition, in many pre-clinical models of inflammation and autoimmunity, GM-CSF neutralization or deletion suppresses a number of diseases including the following: arthritis, experimental autoimmune encephalomyelitis, lung inflammation and disease (asthma and chronic obstructive pulmonary disease), nephritis, psoriasis, atherosclerosis, cancer (e.g., breast cancer), Alzheimer’s disease, myocardial infarction, peripheral insulin resistance, and inflammatory pain. Thus, beneficial effects of GM-CSF-driven tolerance in CD seem somewhat counterintuitive, as GM-CSF is generally regarded as a cytokine with more proinflammatory functions based on its activity on neutrophils and macrophages. It is likely that GM-CSF plays both protective and pathological roles in IBD and that the context in which it is produced might determine its ultimate functional role. Recent work, for example, showed that GM-CSF promotes IL-23-driven intestinal inflammation through local accumulation of activated eosinophils and potentiation of their effector functions [40]. A targeted cell-based approach using GM-CSF-activated monocytes represents a safer and more promising treatment option given the pleiotropic and proinflammatory effects of GM-CSF on inflammation and autoimmunity (reviewed in [41]). Given the contrasting roles for GM-CSF in immunopathology, our work helps to strengthen the link between GM-CSF and IBD [42] and is opening up new possible avenues for a personalized IBD treatment.
